# Wrapped into sound: Development of the Immersive Music Experience Inventory (IMEI)

**DOI:** 10.3389/fpsyg.2022.951161

**Published:** 2022-09-16

**Authors:** Yves Wycisk, Kilian Sander, Reinhard Kopiez, Friedrich Platz, Stephan Preihs, Jürgen Peissig

**Affiliations:** ^1^Institute for Musicology, Hanover University of Music, Drama, and Media, Hanover, Germany; ^2^Institute for Musicology, Music Pedagogy and Aesthetic, State University of Music and Performing Arts Stuttgart, Stuttgart, Germany; ^3^Institute of Communication Technology, Leibniz University Hanover, Hanover, Germany

**Keywords:** music, immersion, emotion, 3D audio, psychometrics, many-facet Rasch measurement, item response theory

## Abstract

Although virtual reality, video entertainment, and computer games are dependent on the three-dimensional reproduction of sound (including front, rear, and height channels), it remains unclear whether 3D-audio formats actually intensify the emotional listening experience. There is currently no valid inventory for the objective measurement of immersive listening experiences resulting from audio playback formats with increasing degrees of immersion (from mono to stereo, 5.1, and 3D). The development of the Immersive Music Experience Inventory (IMEI) could close this gap. An initial item list (*N* = 25) was derived from studies in virtual reality and spatial audio, supplemented by researcher-developed items and items extracted from historical descriptions. Psychometric evaluation was conducted by an online study (*N* = 222 valid cases). The *N* = 222 Participants (female = 112, mean age = 38.6) were recruited via mailing lists (*n* = 34) and via a panel provider (*n* = 188). Based on controlled headphone playback, participants listened to four songs/pieces, each in the three formats of mono, stereo, and binaural 3D audio. The latent construct “immersive listening experience” was determined by probabilistic test theory (item response theory, IRT) and by means of the many-facet Rasch measurement (MFRM). As a result, the specified MFRM model showed good model fit (62.69% of explained variance). The final one-dimensional inventory consists of 10 items and will be made available in English and German.

## 1 Introduction

In the early days of sound transmission and reproduction, one of the main technological aims was the rendition of spatial concert atmospheres over loudspeakers (Boren, 2018). In the 1950s, when stereo media hit the market (Geluso, 2018), a 2-channel recording and reproduction system seemed to be the landmark for high-fidelity playback of music. However, as early as 1940, and in cooperation with the conductor Leopold Stokowski, the entertainment industry initiated the application of rear and elevated speakers for the Walt Disney film “Fantasia” (1940) and initiated the new spatial audio format Fantasound (Boren, 2018). This even had a “voice of God” loudspeaker mounted on the ceiling (Rumsey, 2018). In the following decades, a variety of technological developments was necessary to accomplish the evolution from monophonic to 3D sound reproduction with the main aim of creating a spatial illusion. Most of the technical approaches, however, were limited to the listening experience of surround sound in the horizontal plane (Kim, 2018). In the 1970s, Granville Cooper and Michael Gerzon played a key role in the further development of 3D audio formats. Based on a recording and playback system with four stereo channels, Cooper sought to recreate a concert performance in the home environment (Cooper, 1970). This system was called tetrahedral ambiophony and could fulfill the basic psychoacoustic requirements for a three-dimensional sound field construction with a limited number of four loudspeakers in front, rear, and elevated positions (Cooper, 1970; Nicol, 2018; Zotter and Frank, 2019, p. 10; Gerzon, 1971). Since then, additional audio formats such as Auro 3D, Dolby Atmos and DTS:X have been developed. All of the aforementioned playback technologies can be summed up under the terms “3D audio” or “immersive audio.” *Immersive audio* describes the “psychological sensation of being surrounded by specific sound sources as well as ambient sound” (Wenzel et al., 2018, p. 5). Binauralizations over headphones and multi-channel technologies over loudspeakers can be used to elicit this sensation (Wenzel et al., 2018). Both approaches have disadvantages that may reduce the spatial impression. In headphone playback Head-Related Transfer Function (HRTF) mismatches between subjects and binauralizations can occur. Additionally, when compared to generalized HRTFs (as implemented in most binaural renderers), individualized HRTFs can improve perceptual attributes such as sound localizability, externalization, and realism (Jenny and Reuter, 2020). Unfortunately, there is currently no standardized procedure for implementing individualized HRTFs into internet-based studies with large sample sizes. In loudspeaker playback, the localization of virtual (phantom) sound sources in the median plane between vertical loudspeakers are problematic because localization mechanisms based on time and level are insufficient and spectral cues may be ambivalent (Pulkki, 2001).

However, the question remains as to whether there is a relationship between the increasing spatiality of sounds and the listener’s emotional response. Research on the assessment and evaluation of multichannel stimuli, sound systems, and spatial audio in general already exists (Rumsey, 1998; Zacharov and Pedersen, 2015; Francombe et al., 2017a,b). These studies focus mainly on the quality of sound reproduction, attribution, and listener preferences. In contrast, our study is aimed at the perception of sound and the participants’ listening experience. We are interested in the quantification of the extent to which the psychological latent construct of immersion is affected by stimuli in regard to the playback format. Valid high quality psychometric inventories are therefore utilized. By using the Geneva Emotional Music Scale (GEMS; Zentner et al., 2008), Hahn (2018) conducted a first approach to measuring emotions evoked by 3D audio, surround sound, and stereo. However, the latent construct of immersion could not be investigated by the GEMS inventory. According to Görne (2007, p. 89), the goal of a stereo recording is to place the listener in a virtual acoustic environment. One characteristic of a successful recording is the impression of a virtual space. Following this line of reasoning, a comparison of audio playback formats should be based on the extent to which a listener feels immersed in a virtual acoustic environment (e.g., in stereo, surround sound, and 3D audio). This presupposes an objective tool for measurement that is currently unavailable. The only two inventories that come closest to our research question focus either on the perceptual evaluation of spatial audio technologies (Lindau et al., 2014) or on the development of a consensus vocabulary (and its application) for the perceptual space of venues for music and speech performance (Weinzierl et al., 2018).

In this context, the key term *immersion* is an important concept from virtual reality research, which can be “characterized by diminishing critical distance to what is shown and increasing emotional involvement in what is happening” (Grau, 2003, p. 13). Other related terms to the conceptual field of immersion are *absorption* and *presence*, for which a variety of partially overlapping definitions exist. For example, absorption is defined as “an extreme involvement or preoccupation with one object, idea, or pursuit, with inattention to other aspects of the environment. […]” (VandenBos, 2015, p. 4), and presence is understood as “the subjective experience of being in one place or environment, even when one is physically situated in another” (Witmer and Singer, 1998, p. 225). For the immersion-related term of presence, the most concise definition is the experience of “being there” (Lombard and Jones, 2015, p. 16). Some studies also assume the existence of social presence. For example, Shin et al. (2019) found evidence that 3D sound can play a key role in triggering social presence, thereby positively influencing enjoyment. However, due to the lack of clear definitions and comprehensive concepts, a plain distinction between the various types of presence is difficult. We define immersion as a “psychological state characterized by perceiving oneself to be enveloped by, included in, and interacting with an environment that provides a continuous stream of stimuli and experiences” (Witmer and Singer, 1998, p. 227). In this context, being immersed means being involved in a given context, not only physically but also mentally and emotionally (Georgiou and Kyza, 2017). For our study, we further assume as a working definition that immersion is a continuous latent trait. Its manifestation may be dependent on innate and learned hearing mechanisms. We presume that psychoacoustic and electrophysiological correlates exist.

Although inventories for the operationalization of these terms already exist, they are predominantly related to the visual domain. These selected existing inventories will serve as a starting point for the development of an audio-specific inventory (see Table 1).

**TABLE 1:** Initial list of 25 items plus two additional control items (C1 and C2).

#	German	English	Source/inspired by
1	Mir gefiel das Zuhören, da es für mich ein neuartiges Hörerlebnis war.	I enjoyed listening, as it was a new kind of listening experience for me.	Georgiou and Kyza, 2017
2	Ich habe gerne Zeit zum Zuhören aufgewendet.	I enjoyed spending time listening.	Georgiou and Kyza, 2017
3	Ich hätte das Musikstück gern bis zum Ende gehört.	I would have liked to carry on listening till the end of the piece/song.	Georgiou and Kyza, 2017
4	Ich mochte das Zuhören.	I enjoyed listening.	Georgiou and Kyza, 2017
5	Das Hörerlebnis fesselte mich.	The listening experience captivated me.	Georgiou and Kyza, 2017
6	Ich empfand das Zuhören oft als aufregend.	I often found the music exciting to listen to.	Georgiou and Kyza, 2017
7	Ich war neugierig auf den weiteren Verlauf des Hörerlebnisses.	While I was listening, I was curious as to how the experience of listening would continue.	Georgiou and Kyza, 2017
8	Ich war oft aufgeregt, weil mich die Musik unmittelbar erreichte.	I was excited because I felt a direct connection with the music.	Georgiou and Kyza, 2017
9	Beim Zuhören verblassten alltägliche Gedanken.	While I was listening, my everyday thoughts faded away.	Georgiou and Kyza, 2017
10	Beim Zuhören verblassten alltägliche Sorgen.	As I listened, my everyday concerns faded away.	Georgiou and Kyza, 2017
11	Beim Zuhören konnte mich kaum etwas ablenken.	While I was listening, hardly anything could distract me.	Georgiou and Kyza, 2017
12	Beim Zuhören verlor ich mein Zeitgefühl.	While listening, I lost all sense of time.	Georgiou and Kyza, 2017
13	Ich dachte, die Musiker würden live vor mir spielen.	It felt like the musicians were playing live, right in front of me.	Georgiou and Kyza, 2017
14	In einigen Momenten wollte ich mit den Musikern mitmachen.	There were moments in which I wanted to join in with the musicians.	Georgiou and Kyza, 2017
15	Das Musikhören war mein einziger Wunsch.	My only wish was to listen to the music.	Georgiou and Kyza, 2017
16	Musik auf diese Weise hören zu können, gefiel mir.	I enjoyed being able to listen to music in this way.	Lindau et al., 2014
17	Beim Zuhören fühlte ich mich von der Außenwelt losgelöst.	While listening, I felt as if I were detached from the rest of the world.	Jennett et al., 2008
18	Mein Hörerlebnis entsprach weitgehend meiner Hörerfahrung im Konzert.	My listening experience was very similar to attending a live concert.	Witmer et al., 2005
19	Das Hörerlebnis war überwältigend.	My listening experience was overwhelming.	Reger, 1982
20	Beim Musikhören fühlte ich mich ,,an die Wand gedrückt“.	I felt “blown away” as I listened to the music.	Reger, 1982
21	Das Hörerlebnis hat mich stark berührt.	The listening experience moved me.	Reger, 1982
22	Die Musik schien losgelöst von den Lautsprechern/Kopfhörern.	The music seemed detached from the loudspeakers/headphones.	Wagner, 2004
23	Von überall her erklang die Musik.	The music resounded from everywhere.	Wagner, 2004
24	Ich fühlte mich an den Ort der Darbietung versetzt.	I felt transported to the actual performance.	Hartmann et al., 2016
25	Aus klanglicher Hinsicht war es ein überzeugendes Hörerlebnis.	In terms of sound, it was a convincing listening experience.	RD
C1	Das Musikstück hat mir gefallen.	I enjoyed the piece of music.	RD
C2	Ich hatte bei diesem Musikstück ein dreidimensionales Hörerlebnis.	I had a three-dimensional listening experience while listening to this piece/song.	RD

RD, researcher-developed item.

### 1.1 Study aims

The main aim of the study was the development of an inventory for the measurement of subjectively perceived degrees of auditory immersion. In the future, this would allow, for example, the comparison of immersive experiences resulting from different audio playback formats. For this purpose, a multi-stage process for test development was used (Irwing and Hughes, 2018) that comprised reviewing the theoretical background, selecting and generating items, and evaluating items based on psychometric criteria. As the development of an inventory requires a large number of participants (in our case, *N* > 200), a laboratory study seemed to be unrealistic. For this reason, we decided to use a web-based approach by conducting an internet experiment. Because most participants would not meet the technical requirements for the standards of 3D audio playback via loudspeakers (e.g., elevated or upfiring speakers), binaural 3D versions of musical stimuli had to be selected or created so that a 3D effect could be generated by means of headphones.

As the perceived 3D effect in binaural productions is influenced by many factors, for example, the individual HRTF (Poldy, 2001), the selection of the stimuli remained a particular challenge. To generate a sufficient amount of response variance, we had to confirm that the binaural 3D audio material had the potential to elicit a convincing 3D effect among the participants. This was to be guaranteed by extensive pre-testing and additional evaluation of the auditory stimuli through experienced sound engineers.

Following data collection, advanced psychometric routines such as confirmatory factor analysis (CFA) and item response theory (IRT) were applied so that we could decide on the dimensionality of the latent construct immersion and the validity and reliability of items (Brown, 2018). In the end, a short inventory (with a length of about 10 items) was to be made available to the research community for future evaluation of listening situations in which spatial audio and immersive audio experiences are of interest.

## 2 Materials and methods

The first step in the development of the Immersive Music Experience Inventory (IMEI) was the wording and selection of items (section “Formulation and selection of items”) to compile a set of candidate items. In a second step, an online study was conducted to acquire data for the psychometric evaluation of these candidate items (section “Online study”).

### 2.1 Formulation and selection of items

A mixed strategy of item identification and item generation was applied: In a first step, a literature review in data bases provided by PsycINFO and ProQuest was conducted on the topics of virtual reality, gaming, and spatial audio focusing on inventories that address the notion of the key terms: immersion, absorption, involvement, or presence. As the majority of inventories came from the domain of augmented or virtual reality research, the wording of selected items had to be refocused to listening. The original items were mainly used as a source of inspiration and had to be adapted significantly. For example, an item such as “I liked the type of the activity” (Georgiou and Kyza, 2017) was reformulated to “I enjoyed listening,” and an item such as “I felt detached from the outside world” (Jennett et al., 2008) was adapted to “While listening, I felt as if I were detached from the rest of the world.” Additionally, items extracted from historical descriptions of spatial audio effects (Items 22 and 23) and researcher-developed items were added (Items 25, C1, and C2). On this basis, an initial item set of 25 candidate items and two control items was compiled (see Table 1). For the original wording of items and their adaption, see Supplementary Table 1.

The wording of the items was meant to capture the personal listening experience (emotions felt) and not offer a description of the technical properties of the sound or music or what it conveys (emotions perceived). Therefore, items were predominantly formulated as first-person statements. In addition, items related to hypothetical situations, performance or learning tasks, control of the situation, or visual aspects were disregarded. In the case of items with similar content from different inventories, the item that could be adapted best to music perception was selected. Identical items from different sources were only considered once.

After the selection and adaptation process, a German and an English version of the initial item set was created according to the standards of cross-cultural research methods and test adaptation (van de Vijver and Leung, 2011; Tran et al., 2017; e.g., translation, evaluation, and retranslation; International Test Commission, 2017). Table 1 contains all items from the initial list and two additional items to control for the liking of the piece/song and for the impression of three-dimensionality. A 4-point rating scale with labeled extremes [1 = *strongly disagree* (*Trifft ganz und gar nicht zu*), 4 = *strongly agree* (*Trifft voll und ganz zu*)] was used for item responses.

### 2.2 Online study

An online study was conducted for the psychometric evaluation of the German version of the item set from 28 January to 2 March 2021 using the platform *SoSci Survey*^1^. All standards for the implementation of an internet listening experiment, such as high hurdle techniques or a check of participants’ audio equipment, were considered (Reips, 2012). In terms of sample size, according to classical recommendations on sample size for exploratory factor analysis (EFA), a sample-to-subject ratio of about 10: 1 can be regarded as a reasonable starting point (Osborne, 2014). This results in a sample size of about 250 valid cases for the EFA. Due to the expected high demands on participants’ endurance and audio equipment, this seemed to be a realistic target sample size. Finally, for the scheduled many-facet Rasch measurement (MFRM) model, a minimum of 30 observations per element (e.g., a participant or an item) and at least 10 observations per response scale category (4-point) were necessary for stable estimates of the respective parameters (Linacre, 2021a), achieved with the sample size required for factor analysis.

#### 2.2.1 Stimuli

Potentially suitable audio material was gathered from a variety of sources. Due to the general methodological approach, mono, stereo, and 3D versions of all pieces were required. As an online study was to be conducted, all 3D audio samples had to be available as binaural versions for headphone usage. In general, three different approaches were used to create the final binaural headphone versions for each piece/song: (a) extraction of original binaural 3D releases from CDs and BDs; (b) production of original 3D mixes with Dear Reality dearVR MUSIC (Version 1.40^2^); (c) transformation of 3D audio material intended for loudspeaker playback by using the Dolby Atmos Renderer (Version 3.4) from the Dolby Atmos Production Suite^3^.

Through an extensive iterative process of external and internal evaluation of the stimuli regarding their degree of immersion, four suitable pieces/songs were selected (for the final stimulus list see Supplementary Table 2). Based on these four preselected 3D stimuli, three audio engineering experts identified the respective section of every piece/song with the strongest 3D effect. Stereo and mono versions were added to the stimulus selection as additional formats with predictable lower degrees of immersion. For the online study, all stimuli were normalized to −20 LUFS (integrated). The length of each section was about 60 s and was kept constant across all versions of a piece/song. This length is considered to be sufficient as the mean initial emotional response time to audio stimuli is around 8.31 s (Bachorik et al., 2009). Our sample duration exceeded this minimum requirement. All sound examples were presented in wav format. Details of the complete stimulus selection process are described in Supplementary Figure 1.

#### 2.2.2 Procedure

**FIGURE 1:**
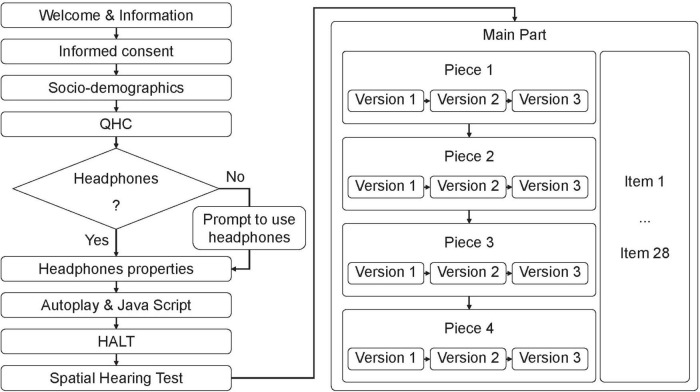
Flowchart of the online study. QHC, Quick Hearing Check; HALT, Headphone and Loudspeaker Test. Items and versions were presented in random order. For example, Version 1 of Piece 1 could be presented in mono whereas Version 1 of Piece 2 could be in 3D. Items 1–28 consisted of the 25 candidate items, the two control items, and the instructed response item.

Figure 1 depicts the entire procedure of the online study. On the welcome page of the survey, participants were informed that the study was about music perception and that participation would take about 45 min. Information on technical requirements was given (e.g., audio playback equipment and deactivation of sound processing enhancements of the operating system). All attendees were informed that various tests on attentive participation would be embedded and that response time would be recorded. The informed consent of the participants was then requested.

To check for participants’ attention, we administered a short calculation task (4 + 5 = ?). Additionally, the input field was not limited to the character length of the solution. This task was to exclude those participants using autofill scripts for the completion of questionnaires. The same filter criterion was applied to the input field in which participants were asked to state their age. Next, participants indicated their gender, educational level according to the ISCED (UNESCO, 2012), and whether they were in a music-related profession.

The *Quick Hearing Check* (QHC; Kochkin and Bentler, 2010) is a 15-item self-report on hearing loss. According to the QHC instructions, sum scores of 32 or higher generally indicate a severe hearing loss; this functioned as an exclusion criterion in our study. An instructed response item was embedded in the list of original items of the QHC to detect participants who produced meaningless data by non-attentive response behavior (Leiner, 2019).

Next, participants had to indicate the kind of playback device they used in this study from a list of playback devices (i.e., headphones, built-in laptop, smartphone, or tablet speaker(s), speakers in a monitor/TV, or freestanding speakers). Self-reported non-headphone users were informed that the use of headphones was mandatory for this experiment, and usage would be controlled by listening tasks. In the next step, the type (circumaural, supra-aural, and intra-aural), the manufacturer, and the model of the headphones used had to be provided. Next it was checked whether autoplay and Java Script were enabled in the browser. For several browser types, brief instructions on how to set up requirements were given. Windows users were instructed to deactivate all sound processing enhancements.

After the technical requirements were established, participants completed the Headphone and Loudspeaker Test (HALT; Wycisk et al., 2022a,b). HALT comprises tasks for calibrating the playback level, checking the correct assignment of stereo channels, estimating the lower cutoff frequency, and screening for headphone usage. In the original HALT laboratory experiment with various playback devices, participants set an average level of 67.77 dB(A) (test-retest reliability *r_*tt*_* = 0.899) with a relatively low heterogeneity (SD = 4.29) by using a counting task. The subjectively adjusted sound pressure level was measured with a short section from a pop song (long term LUFS = −8.4). The test-retest reliability of HALT for detecting mono/stereo playback (*r*_*tt*_ = 0.821) and for detecting the lower frequency limit (*r*_*tt*_ = 0.792) is high (Wycisk et al., 2022a). As headphones of different quality were used in the HALT study, we expect a similar setting and reliability of the volume standardization in the inventory development for the IMEI. To maximize the percentage of correct classifications, HALT can determine the optimal scoring for the screening procedure for a given prevalence, that is, the proportion of headphone users in the target population. Therefore, the assumption was made that 75% of the participants who reported using loudspeakers switched to headphone use after receiving instructions to use headphones. HALT comprises three individual playback device screening tests A, B, and C (the latter developed by Woods et al., 2017). For a prevalence of 75% it would optimal to use all three tests according to the utility-driven approach described by Wycisk et al. (2022b) in the following way: A Participant has to pass test C and at least one of tests A and B to be classified as a headphone user. To pass a screening test a participant needs a minimum of 6, 3, and 5 correct responses for test A, B, and C, respectively.

As a manipulation check (perception of differences between the audio formats), a spatial hearing test (comparison task, 2-AFC design) was used: Participants listened to three pairs of sound samples and decided which sound sample of a pair showed higher spatiality. Pairs and pair positions were presented in random order and based on the same 20-s excerpts used for the main study (rendered either in mono or in 3D audio). One pair served as a retest item.

After the participants completed the initial tests, the main part of the study started. A complete (fully crossed) design was used (Eckes, 2015, p. 153). Because there are no missing values, this design leads to the highest precision of model parameter estimates. In our study, all items were presented in random order. To reduce cognitive load, we defined and kept constant a random order of the candidate items and control items for each participant throughout the entire procedure. Instructed response items were embedded between the original candidate items for each stimulus, which enabled us to check for attentive participation. The stimuli were randomized in two steps for each participant: First, the order of versions (mono, stereo, and 3D) was randomized for each piece/song. Second, the pieces/songs were placed in random order. Each stimulus was, first, automatically played on a blank questionnaire page. After the stimulus had been played once completely, the candidate items and control items were displayed with their 4-point rating scale along with control buttons for replaying and pausing the stimulus.

Additional criteria for data trimming were predefined to ensure data quality: In case of two incorrectly answered instructed response items, the participant was excluded from the survey. The cases in which participants took longer than 5 min to answer the items for one stimulus were flagged. If a processing duration of 5 min was exceeded a second time for the same case, the flagged participants were excluded from the survey.

#### 2.2.3 Participants

**FIGURE 2:**
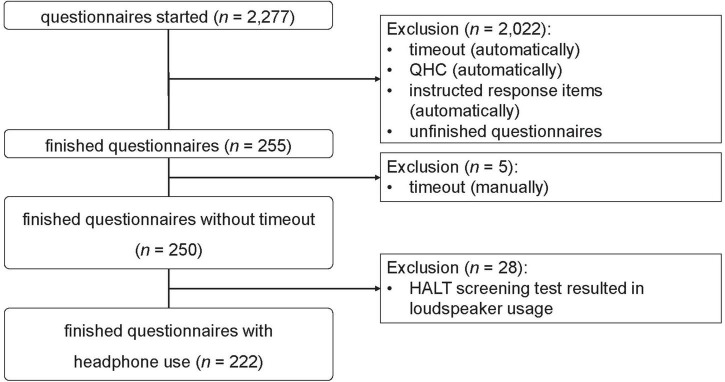
Flowchart of the data filtering process for the online study.

Participants were acquired from a commercial sample provider (mo’web GmbH, Germany^4^) and through target-group specific mailing lists. Multiple criteria for the filtering of meaningless data were applied during data collection. As shown in Figure 2, of *N* = 2,277 commenced questionnaires, only 255 were completed; 2,022 were excluded due to the incorrect answering of the instructed response items, high QHC scores, or dropout. Five participants had to be excluded manually due to repeated timeout. To exclude participants who did not use headphones, we applied the results of the HALT screening procedure. Twenty-eight participants were classified as loudspeaker users and were therefore excluded. The remaining 222 participants comprised the final sample and were the basis for the next steps of data analysis. Table 2 shows socio-demographic data for this sample and the subsamples grouped by type of acquisition.

**TABLE 2:** Descriptive statistics and socio-demographics of the sample.

		Total sample (*N* = 222)	Subsample from provider (*n* = 188)	Subsample from mailing lists (*n* = 34)
Gender*	female	112 (50.5%)	105 (55.9%)	7 (20.6%)
	male	110 (49.5%)	83 (44.1%)	27 (79.4%)
Age	Mean (*SD*)	38.6 (11.8)	39.9 (11.4)	31.7 (12.0)
ISCED 2011 Level	Median (IQR)	4.0 (3.0)	4.0 (3.0)	6.0 (3.0)
	Min–Max	1–8	1–7	3–8
Music-related profession	*n* (%)	38 (17.1%)	13 (6.9%)	25 (73.5%)
QHC score	Mean (*SD*)	5.52 (6.84)	5.26 (6.92)	6.97 (6.29)

IQR, interquartile range. *No participant selected the category of “other” for gender.

#### 2.2.4 Ethical approval statement

The study was performed in accordance with relevant institutional and national guidelines (Hanover University of Music, Drama and Media, 2017; Deutsche Gesellschaft für Psychologie, 2016) and with the principles expressed in the Declaration of Helsinki. Formal approval of the study by the Ethics Committee of the Hanover University of Music, Drama and Media was not mandatory as the study adhered to all required regulations. Anonymity of participants and confidentiality of their data were ensured. They were informed about the objectives and the procedure of the survey as well as the option to withdraw from the study at any time without providing reasons or having any repercussions. All participants gave their informed consent online in accordance with the guidelines of the Hanover University of Music, Drama, and Media, by ticking a checkbox.

## 3 Results

**FIGURE 3:**
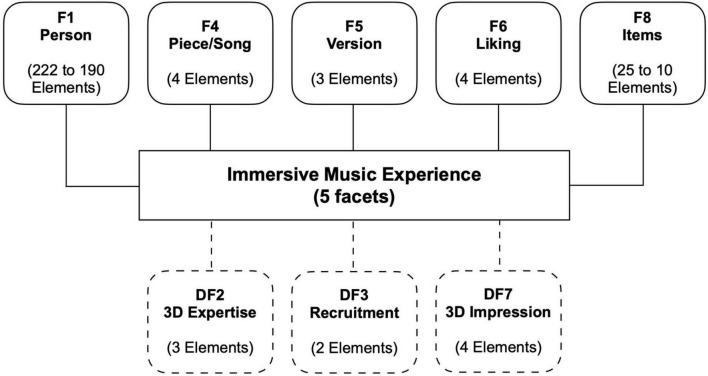
Facet model for the MFRM analysis. F1–F8 represent the five facets of the model and DF2–DF7 the three dummy facets (only considered for interaction effects but not for main effects).

In addition to factor analytical techniques from classical test theory (CTT), approaches from item response theory (IRT) were considered for the psychometric evaluation of the candidate items. In contrast to CTT, an IRT-based approach allows the separation of the influence of individual parameters (e.g., person and items) on the resulting score (van der Linden, 2018). A variety of different influences (context and situation factors) on auditory immersion is to be expected. For the IRT family of methods, the MFRM model is a unidimensional IRT model which allows for inclusion of multiple context or situation factors—known as facets—in addition to the two facets of item difficulty and subject ability considered in the standard Rasch model (Linacre, 1994; Eckes, 2015; Janssen, 2018). MFRM assumes a stochastic relationship between response behavior and a latent dimension. The selected number of facets comprise the model to be tested and are considered in the calculation of IRT indices (e.g., person and item estimates). As shown in Figure 3, the defined MFRM model comprised five facets (F1, F4, F6, and F8) and three dummy facets (DF2, DF3, and DF7). The following descriptions are of the facets:

F1 Person: Two persons listening to the exact same audio can differ in their immersive audio experience. For performance assessment applications of IRT, this difference would be attributed to the ability of the individuals. Here, the responsible person trait could be described as receptivity or propensity for immersion. Each participant is considered as an element of this facet.

F4 Piece/Song: Different songs or pieces might contribute differently to the immersive audio experience. This characteristic could be termed potential for immersion. The four pieces of music used constitute the four elements of this facet.

F5 Version: Analogously, different versions, namely, audio formats, could have different potential for immersion. Mono, stereo, and 3D-audio are the three elements of this facet.

F6 Liking: Different degrees of liking a piece of music might influence the immersive audio experience. The four response categories of item C1 are the elements of this facet.

F8 Items: For the same immersive audio experience, a person might respond differently to several items. This is because some items require more of the latent construct than others to achieve the same (high) response category. This characteristic is represented by the item difficulty. The candidate items constitute the elements of this facet.

We introduced dummy facets (DF) into the model to test for interactions between facets and potentially influencing variables that are not considered as facets in their own right. The following serves as a description of the dummy facets:

DF2 Expertise: Differences in expertise related to music and audio production might influence the immersive audio experience. Participants were assigned to one of three levels of expertise based on their indication of music-related profession. The three levels are the elements that constitute this dummy facet.

DF3 Recruitment: Participants were acquired from a panel provider and via mailing lists. This might have influenced the response behavior. Therefore, the two sources for participants represent the elements of the dummy facet.

DF7 3D Impression: The four response categories of item C2 (from 1 = “*strongly disagree*” to 4 = “*strongly agree*”) are the elements of this dummy facet.

The log odds form of the model without dummy facets is given by


(1) $$\ln\left(\frac{p_{n\,i\,m\,j\,l\,k}}{p_{n\,i\,m\,j\,l\,k-1}}\right)=\theta_{n}-\delta_{i}+\omega_{m}+\xi_{j}+\lambda_{l}-\tau_{k}$$

where *p*_*nimjlk*_ is the probability that a person *n* responded with category *k* ∈ {2,3,4} to item *i* when they listened to the piece of music *m* in the format *j* with a liking of *l*; *p*_*nimjlk–1*_ is the probability that a person *n* responded with category *k-1* to item *i* when they listened to the piece of music *m* in the format *j* with a liking of *l*; τ_*k*_ is the difficulty of responding with category *k* relative to *k-1*. The difficulty δ_*i*_ of item *i* is the point on the latent variable in which Category 1 and 4 were equally probable. ω_*m*_ and ξ_*j*_ are the potential for immersion of piece/song *m* and version *j*, respectively. λ_*l*_ represents the influence of response category *l* of item C1. The unit of all parameters and, therefore, of the latent dimension is *logits*, that is, log odds units (Eckes, 2015, p. 25).

To test for the assumed unidimensional structure and the role of other influential variables, we applied a principal component analysis of standardized residuals (PCAR; Linacre, 2003; Eckes, 2015, pp. 124–27). Data were preprocessed using Excel and several R packages in RStudio (Version 1.3.959^5^, R, Version 4.0.2^6^, car, Version 3.0-11^7^, dplyr, Version 1.0.5^8^). Exploratory and confirmatory factor analyses were conducted by means of Jamovi (Version 1.6.23^9^). For the main MFRM analysis, the Facets software (Version 3.83.6^10^) was used, and the PCAR was calculated by the software Winsteps (Version 5.0.0^11^).

### 3.1 Exploratory factor analysis of the initial item set

As a test of statistical preconditions, Bartlett’s test of sphericity (χ^2^ = 76209, df = 300, *p* < 0.001) and the Kaiser-Meyer-Olkin measure of sampling adequacy (overall MSA = 0.984, MSA > 0.970 for all candidate items) indicated that the data set was suitable for an EFA (Navarro and Foxcraft, 2019, p. 421). An EFA with varimax rotation and maximum likelihood extraction was performed and revealed that only the first factor (eigenvalue 16.78) showed an eigenvalue greater than 1. Thus, according to the Kaiser-Guttman criterion, only this first factor should be extracted (Moosbrugger and Schermelleh-Engel, 2012). The scree plot was also in favor of just one extracted factor. Although the parallel analysis suggested five factors (model fit: RMSEA = 0.0428, TLI = 0.981, BIC = −372; model test: χ^2^ = 1087, df = 185, *p* < 0.001, total variance explained by the first five factors = 76.1%; see Supplementary Figure 2 for simulated eigenvalues), one should bear in mind that this finding might be the result of psychometrically unsuitable items that disturbed the results (see Supplementary Table 3 for the factor loadings). In general, a comparison of the dimensionality of other immersion-related inventories and the IMEI could not yet be recommended. The dimensionality of an overall immersion as a multisensory phenomenon had not yet been conclusively clarified. Existing hierarchical models assert a cause-and-effect relationship for which no data-based evidence was available. Items from other inventories had to be significantly adjusted in order to meet the needs of the IMEI. When comparing inventories that are evidently different, equality of dimensionality cannot be expected.

### 3.2 Item identification by many-facet Rasch measurement analyses

As the EFA confirmed unidimensionality, in the next step, MFRM analyses were performed. It was assumed that the structure of the 4-point response scale on the latent dimension “immersion” would be the same for all candidate items. Therefore, the rating scale model (RSM) was selected for further analyses rather than the partial credit model (PCM), in which the scale structure would be considered as item-dependent (Eckes, 2015, pp. 27–28; Wright, 1998). The 5 facets participant, item, piece, version (audio format), and liking and the 3 dummy facets expertise, recruitment, and 3D impression were specified (see Figure 3 for the MFRM model). This model was used for an iterative process to determine the final item set (the full process is described in the following paragraph). Based on the two criteria of outfit mean-square statistics and point-measure correlations, outlier participants and items were successively identified and removed from the data set. As a rule of thumb, we decided that no more than 15% of the participants should be excluded as outliers during this process. Generally, mean-square fit statistics indicate the randomness within the probabilistic model and have an expected value of 1.0 (Linacre, 2002). Values smaller than the expected value (model overfit) indicate observations that are too predictable, while values larger than 1.0 indicate too little predictability (model underfit); outfit statistics are outlier-sensitive. Mean-square values were used, rather than standardized fit statistics, because with the latter even small deviations from model expectations become significant in larger samples (Smith et al., 2008). The point-measure correlation provides information on the correspondence between the observed scores and the model expectation (Eckes, 2015, p. 100). Therefore, a negative point-measure correlation indicates poor coincidence of model expectations and observations.

In this section, we describe the iterative procedure for item identification (see Supplementary Figure 3 for a visualization of the procedure). The first step of analysis included the complete data set containing all participants (*N* = 222) and all candidate items (*N* = 25). The item outfit mean-square values ranged from 0.76 to 3.65, and the Rasch measures explained 55.08% of the variance. To identify potentially disturbing outlier participants, we chose the criterion of an outfit mean-square value >1.75, which is below the rule of thumb of 2.00 (Wright and Linacre, 1994) and above the recommended sample size-based threshold for dichotomous models of 1.16 (Wu and Adams, 2013). As a consequence, 20 participants showed an outfit value of >1.75 and were removed from the data set for the second step of analysis.

In this second step, the analysis of the data set with 91.0% of the participants (*n* = 202) and all candidate items resulted in 55.18% of explained variance, characterized by an outfit range from 0.78 to 3.05 for the items. To detect outlier participants in this step, we used the point-measure correlation. As a consequence, one participant was excluded from further analyses due to a negative correlation.

After removing a first set of outlier participants, we performed the third step of analysis to identify items with poor model fit. Item 20 showed an outfit of 3.08 while all other items had values ranging from 0.78 to 1.54. Thus, Item 20 was removed from the data.

The subsequent fourth step of analysis resulted in 56.84% of explained variance with item outfit values ranging from 0.81 to 1.63. Again, exclusion of outlier participants was in line with the criteria of an outfit of >1.75 (*n* = 9) and negative point-measure correlation (*n* = 2) for the fifth step, with 85.6% of the sample remaining (*n* = 190). After these steps, data trimming based on person misfit was discontinued.

The sixth step of analysis started from this data set adjusted for outliers (*n* = 190 participants) and included 24 of the candidate items. In this iteration, the Rasch measures explained 58.17% of the variance, and item outfit values ranged from 0.81 to 1.55. According to the recommended sample size-based threshold for dichotomous MFRM models, item outfit values should be in the range of 0.94–1.06 (Wu and Adams, 2013). However, these thresholds may be too strict in view of the fact that it was not the first step of analysis (Eckes, 2015, p. 79) and that the data were polytomous rather than dichotomous. Thus, the more lenient criterion of an outfit value of >1.2 was applied to exclude items. According to this criterion, Items 23, 22, 18, 25, 13, 24, and 14 were removed from the item set across seven iterations. In the 13th iteration, the remaining 17 items showed an outfit value between 0.90 and 1.16 and were, thus, considered as psychometrically adequate (see Supplementary Table 4 for details).

### 3.3 Final item set

To compile a short final item set, we considered the content of the items as well as their position on the latent dimension immersion, that is, the item difficulty. The main aim of this last step of analysis was to cover a preferably wide range on the latent continuum based on 10 items but without large accumulations in immediate vicinity. Therefore, quintiles (20% percentiles) of the item difficulty distribution were used. The authors discussed items within each quintile, and two items out of each quintile were selected for the final set.

#### 3.3.1 Analysis of the final item set

##### 3.3.1.1 Internal consistency and confirmatory factor analysis

The final item set showed an excellent internal consistency (Cronbach’s α = 0.967, *SD* = 0.903) for the adjusted data set. The quality of this index of internal consistency was comparable to the quality criteria of an intelligence test (Schermelleh-Engel and Werner, 2012). A CFA of the adjusted data with the 10 final items as indicators on just one factor resulted in fit measures indicating good or at least adequate fit (model fit: CFI = 0.978, TLI = 0.972, SRMR = 0.0163; see Supplementary Tables 5, 6 for details; Keith, 2015, 312).

##### 3.3.1.2 Assessment of the many-facet Rasch measurement model fit

To check whether the outlier adjusted data adequately fit the specified Rasch model, we considered the standardized residuals (Wells and Hambleton, 2018). A reasonable fit is indicated when the mean of the standardized residuals is close to 0 (Wells and Hambleton, 2018, 198; Linacre, 2021a) and their standard deviation near 1 (Linacre, 2021a, 198), which was the case (*M* = −8.15 × 10^–4^, *SD* = 1.01). Furthermore, about 5% or less of the absolute standardized residuals should exceed values ≥ 2, and about 1% or less should have values ≥ 3 (Eckes, 2015, 69; Linacre, 2021a, 178), which was also the case with 4.5% being ≥ 2 and 0.9% being ≥ 3.

##### 3.3.1.3 Many-facet Rasch measurement analysis

**FIGURE 4:**
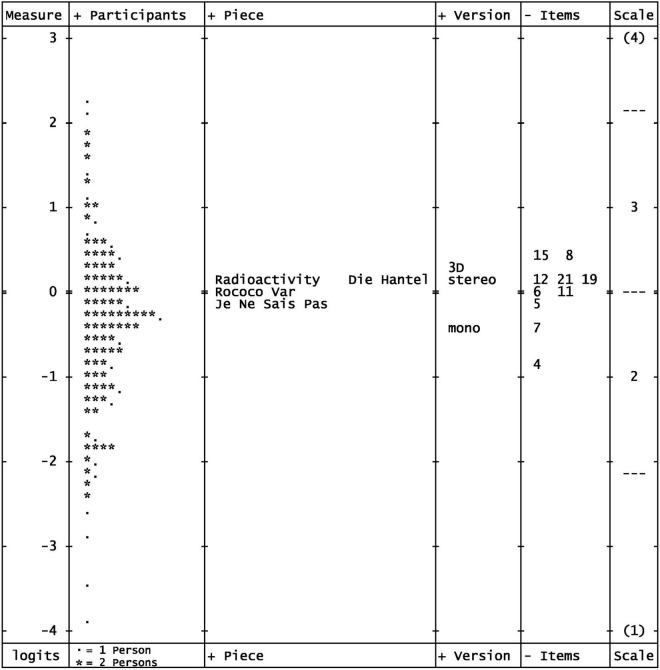
Wright map from the MFRM analysis of the final 10-item set based on the data set adjusted for outliers. *N* = 190 participants. Measure = immersion in logits, “---” in the scale column represents the Rasch-half-point thresholds corresponding to expected values of 0.5 score points.

The characteristics of the model as an outcome of iterative MFRM analysis can be summarized in five steps as follows: First, the Rasch measures explained 62.69% of the variance. Second, as shown in Tables 3, 4, the outfit values of the items ranged from 0.91 to 1.17 and were, thus, in the targeted range. The position of the items, that is, item difficulty, was almost identical to that from the previous analysis (see Table 3 and Supplementary Table 4) so that a range from −0.81 to 0.40 was covered. Third, Figure 4 shows the resulting Wright map (Wilson, 2011) with the facets of participant, piece, version, item, and the (4-point) response scale. As expected, the 3D format was localized slightly higher (0.26 logits) on the latent continuum of immersion than the stereo format (0.14 logits), which was more distinct from the mono format (−0.40 logits; see Supplementary Table 7 for the detailed measurement report of this facet). This means that a 3D audio version was more likely to be rated higher on the immersion scale than the same sound example in stereo or mono format. This finding supports the assumption that 3D audio formats are more likely to actually trigger an increased immersion experience. Fourth, on the piece/song level, comparison of ratings showed only small differences with regard to their localization on the latent dimension (see Supplementary Table 8 for the detailed measurement report of this facet). Therefore, it could be concluded that the experience of immersion was independent of song genre. Fifth, as shown in the category probability curves (Supplementary Figure 4), the response categories of the rating scale (from 1 to 4) were in the correct order. The Rasch-Andrich thresholds, which represent the transition points where adjacent response categories are equally likely to be observed, were separated each by 2 logits from each other so that no collapsing of categories was necessary (Eckes, 2015, 121; see Supplementary Table 9 for details on the response scale category statistics).

**TABLE 3:** Measurement report for the final 10-item set of the *Immersive Music Experience Inventory trimmed* for outliers.

Item	Total Score	Observed Average	Fair (M) Average	Measure (logits)	Model SE	Outfit		Correlation	
	
						MnSq	ZStd	PtMea	PtExp
15	5146	2.26	2.08	0.40	0.03	0.91	−2.4	0.79	0.77
8	5173	2.27	2.10	0.37	0.03	0.95	−1.4	0.78	0.77
12	5326	2.34	2.19	0.21	0.03	1.00	0.1	0.77	0.77
21	5344	2.34	2.20	0.19	0.03	0.97	−0.7	0.79	0.77
19	5435	2.38	2.25	0.09	0.03	1.08	2.2	0.76	0.78
6	5452	2.39	2.26	0.07	0.03	1.17	4.5	0.75	0.78
11	5542	2.43	2.31	−0.03	0.03	1.16	4.3	0.75	0.78
5	5588	2.45	2.34	−0.08	0.03	0.91	−2.5	0.80	0.78
7	5879	2.58	2.51	−0.40	0.03	1.00	0.0	0.79	0.78
4	6256	2.74	2.73	−0.81	0.03	0.97	−0.8	0.78	0.78

Mean	5514.1	2.42	2.30	0.00	0.03	1.01	0.3	0.78	
*SD*	336.0	0.15	0.19	0.37	0.00	0.09	2.6	0.02	

N = 190. Total Score, observed raw score; Observed Average, observed raw score divided by the number of observations (2,280); Fair (M) Average, Rasch measure to raw score conversion, producing an average rating for the item that was standardized so that it was fair; Measure, item difficulty in logits; Model SE, model standard error; MnSq, mean-square; ZStd, Z-standardized t-statistic; PtMea, point-measure correlation (correlation between the item’s observations and the measures modeled to generate them); PtExp, expected value of the point-measure correlation; SD, standard deviation of the sample (excerpt from Facets Output).

**TABLE 4:** Measurement report and bilingual version of the final 10-item set of the *Immersive Music Experience Inventory*.

#	Item	Measure (logits)	Outfit (Mean-Square)	Source
15	*Das Musikhören war mein einziger Wunsch.* My only wish was to listen to the music.	0.40	0.91	Georgiou and Kyza, 2017
8	*Ich war oft aufgeregt, weil mich die Musik unmittelbar erreichte.* I was excited because I felt a direct connection with the music.	0.37	0.95	Georgiou and Kyza, 2017
12	*Beim Zuhören verlor ich mein Zeitgefühl.* While listening, I lost all sense of time.	0.21	1.00	Georgiou and Kyza, 2017
21	*Das Hörerlebnis hat mich stark berührt.* The listening experience moved me.	0.19	0.97	Reger, 1982
19	*Das Hörerlebnis war überwältigend.* My listening experience was overwhelming.	0.09	1.08	Reger, 1982
6	*Ich empfand das Zuhören oft als aufregend.* I often found it exciting to listen to the music.	0.07	1.17	Georgiou and Kyza, 2017
11	*Beim Zuhören konnte mich kaum etwas ablenken.* While I was listening, hardly anything could distract me.	−0.03	1.16	Georgiou and Kyza, 2017
5	*Das Hörerlebnis fesselte mich.* The listening experience captivated me.	−0.08	0.91	Georgiou and Kyza, 2017
7	*Ich war neugierig auf den weiteren Verlauf des Hörerlebnisses.* While I was listening, I was curious as to how it would continue.	−0.40	1.00	Georgiou and Kyza, 2017
4	*Ich mochte das Zuhören.* I enjoyed listening.	−0.81	0.97	Georgiou and Kyza, 2017

For application purposes when using the IMEI, a 4-point rating scale with labeled extremes [1 = *strongly disagree* (*Trifft ganz und gar nicht zu*), 4 = *strongly agree* (*Trifft voll und ganz zu*)] must be used. For additional statistical details of the items see Table 3.

##### 3.3.1.4 Principal component analysis of residuals

To control for unidimensionality of the 10-item set, we used a PCAR based on the outlier-adjusted data. This revealed contrasts—the principal components—with very similar eigenvalues smaller than 1.6, such that each component had a strength of less than two items (Linacre, 2021b, pp. 416–21; see Supplementary Table 10). Moreover, the Rasch measures of the items and persons each explained more than two and a half times as much variance as one of the contrasts. Another indicator for the unidimensionality was the high correlation of person measures obtained from clusters of items formed according to their loadings on the components of the PCAR (see Supplementary Tables 11, 12).

### 3.4 Application of the Immersive Music Experience Inventory

For the useful application of the IMEI, scoring is necessary to express the individual answers to the items in one overall value. The scale of the inventory allows response values from 1 to 4. By taking the mean of the answers of all 10 items, a possible overall score from 1 to 4 will result in steps of 0.1. To check the admissibility of this scoring procedure in our study, a one-tailed Pearson correlation between the averaged IMEI sum score across all stimuli and the person characteristics (logits) was calculated. A high correlation between the two features of *r*(188) = 0.878, 95% CI [0.847,1.0] was observed. The scatterplot shows a slightly s-shaped arrangement of the data points for items obtained with IRT methods (for details see Supplementary Figure 5). A simple score calculation by averaging the individual response values of the 10 items without complex individual weighting of items was, therefore, considered permissible.

## 4 Discussion

We successfully developed the IMEI for the measurement of immersive music experience with high psychometric quality. The manageable number of ten items allows for an efficient application in multiple research fields in which audio content plays an important role, such as research in the entertainment industry or virtual reality. Possible limitations of our findings should be considered and might have resulted from the use of binaural headphone mixes as 3D stimuli (instead of loudspeaker playback). In the current state of our research, we cannot rule out that the presentation by headphones might underestimate the “true” impact of 3D audio on immersion. However, the question of the magnitude of the effect size will be subject to forthcoming research. Another possible influencing factor on the strength of the 3D effect could result from the mismatch of HRTFs. The HRTFs used in the stimuli are based on average HRTFs of a large sample of listeners but do not match the individual HRTF of a participant. This can result in a suboptimal localization of phantom sound sources. A poor localization could result in an attenuation of the experience of immersion. Furthermore, even a matching HRTF cannot preclude an inappropriate *headphone-to-ear transfer function* (HpTF), which also negatively affects localization. The HpTF is defined as the electroacoustic transfer function of a headphone, measured in the eardrum (Møller, 1992). Differences occur due to interindividual differences in the physiognomy of the pinna. Another uncertainty in the measurement of immersion experiences may result from the differences in bass perception: While a strong bass perception can be a strong bodily sensation in loudspeaker reproduction, this effect is largely absent when the listener uses headphones. However, the binaural approach was pragmatic as the required high number of participants was unrealistic for a laboratory study. In a future laboratory study, the authors will further evaluate the IMEI by using anchor stimuli from the online study in a loudspeaker setup. This will allow direct comparison between binaural 3D audio for headphones and for loudspeakers with the same audio material.

We are also aware that the binaural 3D realizations we used are not the only possible ones: Current state of the art production tools for 3D audio (e.g., dearVR MUSIC, Dolby Atmos Renderer) allow for a number of degrees of freedom in the adjustment of output parameters such as HRTF types and spatial settings. Based on multiple evaluations of the output, we tried to identify the best possible examples of the binaural approach. Although these sources of variation should be considered as sources of uncertainty in measurement, it seems unlikely that such intervening variables will influence the main effect of differences in immersion experience between the three audio formats mono, stereo, and 3D audio. The use of recordings based on Ambisonics (Nicol, 2018; Zotter and Frank, 2019) may be of interest for future studies. However, as we did not produce our own recordings, but materials were extracted from existing recordings (e.g., BDs or other multi-track recordings), we had no influence on the recording technology. In other words, the existing materials’ technology was not adequate for use in binaural renderings based on Ambisonics. This was one of the reasons why the Dolby Atmos Renderer was used in addition to dearVR MUSIC from Dear Reality. Furthermore, the renderer implemented in the Dolby Atmos Production Suite represents an industrial standard.

Finally, the psychometric quality of the identified unidimensional IMEI scale should be considered. Concerning the question of validity, we first refer to content validity: As the majority of items were derived from previous research (see Table 1), items used for the construction of the initial IMEI item list were the result of multiple selection and evaluation processes by previous research in the field of virtual reality. Thus, it seems reasonable to assume that item content reflects the definition of the target construct immersion and is the result of careful selection by expert judges (Abell et al., 2009, p. 103; Sireci and Sukin, 2013; Hughes, 2018). Additionally, the Rasch model itself provides evidence about construct validity: The two major threats of construct validity are construct-irrelevance and construct underrepresentation (Sireci and Sukin, 2013; Hughes, 2018) which are indicated by misfitting items and large gaps in the coverage of the latent dimension by the items, respectively (Baghaei, 2008). Within the iterative MFRM analysis process, we discarded misfitting items and selected ten items from the remaining ones that were located optimally on the latent dimension to cover a wide range, which further supports construct validity. The high correlation of the IMEI score with the 3D impression measured by item C2 (Spearman’s ρ (2,280) = 0.718, *p* < 0.001; see Supplementary Figure 5) could be regarded as first evidence for convergent validity. However, this finding should be interpreted with care as both variables were measured using the same method (Hughes, 2018). Future research will have to consider additional forms of convergent and discriminant validity.

The last criterion of psychometric quality is the reliability of the scale. First, we can refer to the aspect of internal consistency as indicated by Cronbach’s alpha value. This should have a value of α > 0.70 (Abell et al., 2009, p. 94). For the IMEI 10-item scale, we found a value of α = 0.967 (*SD* = 0.903), which is an excellent value. Based on Cronbach’s alpha, we calculated the standard error of measurement (SEM; Abell et al., 2009) as a second reliability index. This value describes the expected variation of the true scores and is an estimate of the standard deviation of the errors of measurement. It should be about 5% or less of the range of possible scores (Abell et al., 2009, pp. 95–96). The obtained SEM for the IMEI 10-item scale was 0.164. This is a reasonably small value of 5.47% in the range of possible scores. Therefore, it is close to the critical value of 5% on a 4-point rating scale. The value of SEM = 0.164 can also be regarded as a 95% confidence interval for all values on the IMEI scale with a 95% CI of ± 0.328 (2 × 0.164). Considering the psychometric properties of the developed inventory, we are looking forward to future applications of this measurement instrument in the research field of immersive audio experience.

## Data availability statement

The datasets presented in this study can be found in online repositories. The names of the repository/repositories and accession number(s) can be found below: Open Science Framework (OSF): https://osf.io/ysvca/.

## Ethics statement

Ethical review and approval was not required for the study on human participants in accordance with the local legislation and institutional requirements. The patients/participants provided their written informed consent to participate in this study.

## Author contributions

YW, KS, RK, and FP conceived the study, conducted the data analysis, and wrote the manuscript. SP and JP conceived the study. All authors contributed to the article and approved the submitted version.

## Footnotes

1
www.soscisurvey.de

2
https://www.dear-reality.com/products/dearvr-music

3
https://www.avid.com/de/plugins/dolby-atmos-production-suite

4
https://www.mowebresearch.com/

5
https://www.rstudio.com

6
https://cran.r-project.org

7
https://cran.r-project.org/package=car

8
https://cran.r-project.org/package=dplyr

9
https://www.jamovi.org/

10
https://www.winsteps.com/facets.htm

11
https://www.winsteps.com/winsteps.htm
