# Supplementary material for: Wrapped into sound: Development of the Immersive Music Experience Inventory (IMEI)
Source: Front Psychol. 2022 Sep 16;13:951161. doi: 10.3389/fpsyg.2022.951161 (PMC9524455; doi:10.3389/fpsyg.2022.951161)
Supplement: Supplementary file 1 [file Data_Sheet_1.pdf]

Supplementary Material

Wrapped Into Sound: Development of the *Immersive Music Experience Inventory* (IMEI)

DOI of the corresponding paper: 10.3389/fpsyg.2022.951161

Yves Wycisk[1], Kilian Sander[1], Reinhard Kopiez[1*], Friedrich Platz[2], Stephan Preihs[3], Jürgen Peissig[3]

[1]Hanover University of Music, Drama, and Media, Institute for Musicology, Hanover, Germany

[2]State University of Music and Performing Arts Stuttgart, Institute for Musicology, Music Pedagogy and Aesthetic, Stuttgart, Germany

[3]Leibniz University Hanover, Institute of Communication Technology, Hanover, Germany

*** Correspondence:**
Reinhard Kopiez
reinhard.kopiez@hmtm-hannover.de

1 Supplementary Tables

Supplementary Table 1. Candidate items and their original wordings. RD = researcher-developed item.

#	German	English	Original Wording	Source / Inspired by
1	Mir gefiel das Zuhören, da es für mich ein neuartiges Hörerlebnis war.	I enjoyed listening as it was a new kind of listening experience for me.	I liked the activity because it was novel.	Georgiou & Kyza, 2017
2	Ich habe gerne Zeit zum Zuhören aufgewendet.	I enjoyed spending time listening.	I wanted to spend time to participate in the activity.	Georgiou & Kyza, 2017
3	Ich hätte das Musikstück gern bis zum Ende gehört.	I would have liked to carry on listening till the end of the piece/song.	I wanted to spend time to complete the activity successfully.	Georgiou & Kyza, 2017
4	Ich mochte das Zuhören.	I enjoyed listening.	I liked the type of the activity.	Georgiou & Kyza, 2017
5	Das Hörerlebnis fesselte mich.	The listening experience captivated me.	The AR application we employed captured my attention.	Georgiou & Kyza, 2017
6	Ich empfand das Zuhören oft als aufregend.	I often found the music exciting to listen to.	I was often excited since I felt as being part of the activity.	Georgiou & Kyza, 2017

7	Ich war neugierig auf den weiteren Verlauf des Hörerlebnisses.	While I was listening, I was curious as to how the experience of listening would continue.	I was curious how the activity would progress.	Georgiou & Kyza, 2017
8	Ich war oft aufgeregt, weil mich die Musik unmittelbar erreichte.	I was excited because I felt a direct connection with the music.	I was often excited since I felt as being part of the activity.	Georgiou & Kyza, 2017
9	Beim Zuhören verblassten alltägliche Gedanken.	While I was listening, my everyday thoughts faded away.	Everyday thoughts and concerns faded out during the activity.	Georgiou & Kyza, 2017
10	Beim Zuhören verblassten alltägliche Sorgen.	As I listened, my everyday concerns faded away.	Everyday thoughts and concerns faded out during the activity.	Georgiou & Kyza, 2017
11	Beim Zuhören konnte mich kaum etwas ablenken.	While I was listening, hardly anything could distract me.	During the activity, hardly anything could distract me.	Georgiou & Kyza, 2017
12	Beim Zuhören verlor ich mein Zeitgefühl.	While listening, I lost all sense of time.	I often forgot the passage of time during the activity.	Georgiou & Kyza, 2017
13	Ich dachte, die Musiker würden live vor mir spielen.	It felt like the musicians were playing live, right in front of me.	The activity felt so authentic that it made me think that the virtual characters/objects existed for real.	Georgiou & Kyza, 2017
14	In einigen Momenten wollte ich mit den Musikern mitmachen.	There were moments in which I wanted to join in with the musicians.	I was so involved in the activity that in some cases, I wanted to interact with the virtual characters/objects directly.	Georgiou & Kyza, 2017

15	Das Musikhören war mein einziger Wunsch.	My only wish was to listen to the music.	The activity became the unique and only thought occupying my mind.	Georgiou & Kyza, 2017
16	Musik auf diese Weise hören zu können, gefiel mir.	I enjoyed being able to listen to music in this way.	Degree-of-Liking (lower – higher).	Lindau et al., 2014
17	Beim Zuhören fühlte ich mich von der Außenwelt losgelöst.	While listening, I felt as if I were detached from the rest of the world.	I felt detached from the outside world.	Jennett et al., 2008
18	Mein Hörerlebnis entsprach weitgehend meiner Hörerfahrung im Konzert.	My listening experience was very similar to attending a live concert.	How much did your experience in the virtual environment seem consistent with your real-world experiences?	Witmer et al., 2005
19	Das Hörerlebnis war überwältigend.	My listening experience was overwhelming.	„[…] der Psalm muß glänzend gehen, so, daß Alles einfach „umgeschmissen“ wird! […]“	Reger, 1982
20	Beim Musikhören fühlte ich mich „an die Wand gedrückt“.	I felt “blown away” as I listened to the music.	„[…] Die Hörer des Psalms müssen nachher als „Relief“ an der Wand kleben […]“	Reger, 1982
21	Das Hörerlebnis hat mich stark berührt.	The listening experience moved me.	„[…] ich will, daß der Psalm eine niederschmetternde Wirkung bekommt! […]“	Reger, 1982
22	Die Musik schien losgelöst von den Lautsprechern/Kopfhörern.	The music seemed detached from the loudspeakers/headphones.	„ […] wir nannten ihn den »**mystischen Abgrund**«, weil er die Realität von der Idealität zu trennen habe, (...) während die aus	Wagner, 1873/2004

			dem »mystischen Abgrunde« geisterhaft erklingende Musik, gleich den, unter dem Sitze der Pythia dem heiligen Urschooße Gaia's entsteigenden Dämpfen, ihn in jenen **begeisterten Zustand** des Hellsehens versetzt […]“	
23	Von überall her erklang die Musik.	The music resounded from everywhere.	„ […] wir nannten ihn den »**mystischen Abgrund**«, weil er die Realität von der Idealität zu trennen habe, (...) während die aus dem »mystischen Abgrunde« geisterhaft erklingende Musik, gleich den, unter dem Sitze der Pythia dem heiligen Urschooße Gaia's entsteigenden Dämpfen, ihn in jenen **begeisterten Zustand** des Hellsehens versetzt […]“	Wagner, 1873/2004
24	Ich fühlte mich an den Ort der Darbietung versetzt.	I felt transported to the actual performance.	It was as though my true location had shifted into the environment in the presentation.	Hartmann et al., 2016
25	Aus klanglicher Hinsicht war es ein überzeugendes Hörerlebnis.	In terms of sound, it was a convincing listening experience.	-	RD
C1	Das Musikstück hat mir gefallen.	I enjoyed the piece of music.	-	RD
C2	Ich hatte bei diesem Musikstück ein dreidimensionales Hörerlebnis.	I had a three-dimensional listening experience while listening to this piece/song.	-	RD

Supplementary Table 2. List of Four Pieces in Three Audio Formats (Mono, Stereo, 3D Audio) Used for the Evaluation of Items From the Initial List. RP = researcher-produced version, RP* = multi-track version obtained from the composer Felix Thiesen.

		Source of Version		
Piece	Interpreter	Mono	Stereo	3D (binaural)
Radioactivity	Kraftwerk	RP	Kraftwerk. (2017). *3-D: The catalogue* [Album; CD]. Parlophone	Kraftwerk. (2017). *3-D: The catalogue* [Album; Blu-Ray]. Parlophone
Die Hantel	Zweitaktmotor (© Felix Thiesen, 2019)	RP*	RP*	RP* with dearVR Music (Version 1.40) [Computer software]
Je Ne Sais Pas	LFDV	RP	LFDV. (2012). *Je ne sais pas* [CD]. Sony Music.	https://www.youtube.com/watch?v=R9Vx4PaElZA
Rococo Variations (P. Tchaikovsky)	Jakob Spahn, Stephan Frucht, Orchestra Academy of the Bayerisches Staatsorchester	RP	Spahn, J. (Soloist). (2018). *Cello concertos: Tschaikowski & Gulda* [Album; CD]. Hänssler Classic.	RP with Dolby Atmos Renderer (Dolby Atmos Production Suite) based on Spahn, J. (Soloist). (2018). *Cello concertos: Tschaikowski & Gulda* [Album; Blu-Ray]. Hänssler Classic.

Supplementary Table 3. Factor loadings from the EFA of the initial item set for five factors based on parallel analysis. A maximum likelihood extraction method in combination with a varimax rotation was used. Factor loadings below 0.4 have been omitted. Data represent $N = 2,664$ (12 stimuli × 222 participants) observations per item.

Item	Factor 1	Factor 2	Factor 3	Factor 4	Factor 5	Uniqueness
1	0.471	0.544				0.254
2	0.734					0.186
3	0.794					0.176
4	0.732	0.447				0.154
5	0.594	0.472				0.172
6	0.518	0.409		0.489		0.241
7	0.745					0.189
8	0.510			0.578		0.189
9			0.706			0.145
10			0.688			0.146
11	0.493		0.446			0.344
12	0.424		0.557			0.212
13		0.477		0.404	0.554	0.158
14	0.445			0.515		0.326
15	0.570		0.419			0.240
16	0.611	0.583				0.168
17	0.424	0.429	0.556			0.181

18		0.524			0.457	0.237
19	0.441	0.584		0.401		0.194
20				0.426		0.766
21	0.563			0.450		0.209
22		0.625				0.287
23		0.659				0.364
24		0.477		0.428		0.213
25	0.453	0.689				0.228
Eigenvalue	16.78	0.70	0.60	0.26	0.15	

Supplementary Table 4. Measurement output from the 13th iteration of data analysis with 17 items and the outlier adjusted data set. *N* = 190. Total Score = observed raw score; Observed Average = observed raw score divided by the number of observations (2,280); Fair (M) Average = Rasch measure to raw score conversion, producing an average rating for the standardized item so that it was fair; Measure = item difficulty on the latent dimension "immersion" (in logits); Model SE = model standard error; MnSq = Mean-square; ZStd = Z-standardized *t*-statistic; PtMea = Point-measure correlation (correlation between the item's observations and the measures modelled to generate them); PtExp = expected value of the point-measure correlation; SD = standard deviation of the sample (excerpt from Facets Output, Table 7).

Item	Total Score	Observed Average	Fair (M) Average	Measure	Model SE	Outfit		Correlation	
						MnSq	ZStd	PtMea	PtExp
15	5146	2.26	2.09	0.45	0.03	0.91	−2.3	.79	.77
8	5173	2.27	2.10	0.42	0.03	0.95	−1.4	.78	.77
10	5239	2.30	2.14	0.35	0.03	0.99	−0.3	.77	.77
9	5300	2.32	2.17	0.28	0.03	1.00	0.0	.77	.77
12	5326	2.34	2.19	0.26	0.03	0.98	−0.5	.78	.77
21	5344	2.34	2.20	0.24	0.03	0.96	−1.1	.78	.77
17	5400	2.37	2.23	0.18	0.03	1.00	0.0	.78	.77
19	5435	2.38	2.25	0.14	0.03	1.06	1.7	.76	.77
6	5452	2.39	2.26	0.12	0.03	1.16	4.3	.74	.77
1	5473	2.40	2.28	0.10	0.03	1.16	4.2	.74	.77
11	5542	2.43	2.32	0.03	0.03	1.13	3.6	.75	.77
5	5588	2.45	2.34	−0.02	0.03	0.90	−3.0	.80	.78
3	5846	2.56	2.50	−0.30	0.03	1.06	1.6	.79	.78
7	5879	2.58	2.51	−0.33	0.03	0.96	−1.1	.79	.78

16	6087	2.67	2.64	−0.55	0.03	1.09	2.5	.76	.78
2	6148	2.70	2.67	−0.62	0.03	0.99	−0.3	.78	.78
4	6256	2.74	2.74	−0.74	0.03	0.92	−2.3	.79	.77
M	5514.1	2.42	2.30	0.00	0.03	1.01	0.3	.77	
SD	336.0	0.15	0.19	0.37	0.00	0.08	2.3	.02	

Supplementary Table 5. Factor loadings from the CFA of the final item set. *N* = 2,280 observations per item (12 stimuli × 190 participants). SE = Standard error.

Item	Estimate	*SE*	95% CI Lower	95% CI Upper	*Z*	*p*	Standardized Estimate
15	0.874	0.0173	0.840	0.908	50.6	< .001	0.852
8	0.908	0.0169	0.875	0.941	53.9	< .001	0.886
12	0.887	0.0173	0.853	0.921	51.2	< .001	0.859
21	0.910	0.0169	0.877	0.943	53.8	< .001	0.885
19	0.886	0.0171	0.852	0.920	51.7	< .001	0.864
6	0.873	0.0170	0.840	0.906	51.3	< .001	0.859
11	0.831	0.0173	0.797	0.865	48.1	< .001	0.825
5	0.943	0.0168	0.910	0.976	56.0	< .001	0.906
7	0.918	0.0181	0.882	0.953	50.7	< .001	0.854
4	0.851	0.0169	0.817	0.884	50.3	< .001	0.849

Supplementary Table 6. Fit measures from the CFA of the final item set. *N* = 2,280 observations per item (12 stimuli × 190 participants). CFI = comparative fit index; TLI = Tucker-Lewis index; SRMR = square-root mean residual; RMSEA = root mean square error of approximation.

CFI	TLI	SRMR	RMSEA	RMSEA 90% CI Lower	RMSEA 90% CI Upper
0.978	0.972	0.0163	0.0814	0.0755	0.0874

Supplementary Table 7. Measurement report for the facet version (audio format) resulting from the analysis of the final 10 item set, based on the data set adjusted for outliers. 3D = audio format 3D, ST = audio format stereo, MO = audio format mono; Total Score = observed raw score; Observed Average = observed raw score divided by the number of observations (7,600); Fair (M) Average = Rasch measure to raw score conversion, producing an average rating for the item that was standardized so that it was fair; Measure = localization on the latent dimension "immersion" (in logits); Model SE = model standard error; MnSq = mean-square; ZStd = Z-standardized *t*-statistic; PtMea = point-measure correlation (correlation between the item's observations and the measures modelled to generate them); PtExp = expected value of the point-measure correlation; SD = standard deviation of the sample (excerpt from Facets Output, Table 7).

						Infit		Outfit		Correlation	
Format	Total Score	Observed Average	Fair (M) Average	Measure	Model SE	MnSq	ZStd	MnSq	ZStd	PtMea	PtExp
3D	19585	2.58	2.43	0.26	0.02	1.01	0.3	1.02	0.9	.77	.78
ST	19039	2.51	2.37	0.14	0.02	0.98	−0.9	1.00	−0.2	.78	.78
MO	16517	2.17	2.09	−0.40	0.02	1.03	1.7	1.02	1.0	.76	.77
M	18380.3	2.42	2.30	0.00	0.02	1.01	0.4	1.01	0.6	.77	
SD	1636.6	0.22	0.18	0.35	0.00	0.02	1.3	0.01	0.7	.01	

Supplementary Table 8. Measurement report for the facet piece from the analysis of the final 10 item set based on the data set adjusted for outliers. Total Score = observed raw score; Observed Average = observed raw score divided by the number of observations (5,700); Fair (M) Average = Rasch measure to raw score conversion, producing an average rating for the item that was standardized so that it was fair; Measure = localization on the latent dimension Immersion (in logits); Model SE = model standard error; MnSq = mean-square; ZStd = Z-standardized *t*-statistic; PtMea = point-measure correlation (correlation between the item's observations and the measures modelled to generate them); PtExp = expected value of the point-measure correlation; SD = standard deviation of the sample (excerpt from Facets Output Table 7).

						Infit		Outfit		Correlation	
Song	Total Score	Observed Average	Fair (M) Average	Measure	Model SE	MnSq	ZStd	MnSq	ZStd	PtMea	PtExp
Radioactivity	14109	2.48	2.35	0.11	0.02	1.03	1.5	1.03	1.1	.78	.79
Die Hantel	13692	2.40	2.34	0.08	0.02	1.04	2.0	1.04	1.5	.78	.78
Rococo Var	14047	2.46	2.27	−0.04	0.02	1.00	0.0	1.01	0.5	.78	.77
Je Ne Sais Pas	13293	2.33	2.22	−0.14	0.02	0.95	−2.5	0.98	−1.0	.78	.78
M	13785.3	2.42	2.30	0.00	0.02	1.01	0.3	1.01	0.5	.78	
SD	376.1	0.07	0.06	0.12	0.00	0.04	2.1	0.03	1.2	.00	

Supplementary Table 9. Response scale category statistics. Outfit = outfit mean-square values; Threshold = Rasch-Andrich thresholds; *SE* = standard error (excerpt from Facets Output, Table 8).

Category	Absolute Frequency	Relative Frequency	Average Measure	Expected Measure	Outfit	Threshold	*SE*
1	5402	24%	−2.48	−2.46	1.1		
2	6549	29%	−0.72	−0.76	0.9	−1.78	0.02
3	6755	30%	0.61	0.66	1.1	−0.06	0.02
4	4094	18%	2.07	2.04	1.0	1.84	0.02

Supplementary Table 10. Standardized residual variance in eigenvalue units (item information units) from the PCAR of the final 10 item set, based on the data set adjusted for outliers. *N* = 2,280 observations per item; Winsteps Output, Table 23.0.

		Observed		Expected
	Eigenvalue	Percentage of Total Variance	Percentage of Unexplained Variance	Percentage of Total Variance
Total raw variance in observations	33.2077	100.0%		100.0%
Raw variance explained by measures	23.2077	69.9%		69.7%
Raw variance explained by persons	18.8592	56.8%		56.6%
Raw variance explained by items	4.3485	13.1%		13.1%
Raw unexplained variance (total)	10.0000	30.1%	100.0%	30.3%
Unexplained variance in 1st contrast	1.5793	4.8%	15.8%	
Unexplained variance in 2nd contrast	1.3908	4.2%	13.9%	
Unexplained variance in 3rd contrast	1.2759	3.8%	12.8%	
Unexplained variance in 4th contrast	1.1083	3.3%	11.1%	
Unexplained variance in 5th contrast	1.0186	3.1%	10.2%	

Supplementary Table 11. Item clusters from the PCAR.

	1st Contrast	2nd Contrast	3rd Contrast	4th Contrast	5th Contrast
Cluster	Items	Items	Items	Items	Items
1	4, 7	11, 12, 15	6, 7, 8	21	11
2	5, 11, 12, 15, 19, 21	6, 7, 8	4, 15	4, 5, 6, 7, 8, 11, 12, 15	4, 5, 6, 7, 8, 19, 21
3	6, 8	4, 5, 19, 21	5, 11, 12, 19, 21	19	12, 15

Supplementary Table 12. Approximate relationships between the participants' measures obtained from PCAR item clusters. *N* = 2,280 observations per item; Winsteps Output, Table 23.0

		Extreme Person Scores Omitted		Extreme Person Scores Included			
PCA Contrast	Items Clusters	Pearson Correlation	Disattenuated Correlation	Pearson Correlation	Disattenuated Correlation	Cluster Sizes	
1	1 – 3	0.7208	0.9823	0.7996	1.0000	2	2
1	1 – 2	0.8077	0.9916	0.8667	1.0000	2	6
1	2 – 3	0.8295	1.0000	0.8847	1.0000	6	2
2	1 – 3	0.8237	0.9846	0.8864	1.0000	3	4
2	1 – 2	0.8119	0.9954	0.8771	1.0000	3	3
2	2 – 3	0.8577	1.0000	0.9080	1.0000	3	4
3	1 – 3	0.8553	1.0000	0.9073	1.0000	3	5
3	1 – 2	0.8087	1.0000	0.8735	1.0000	3	2
3	2 – 3	0.8158	1.0000	0.8811	1.0000	2	5
4	1 – 3	0.6849	1.0000	0.7660	1.0000	1	1
4	1 – 2	0.8038	1.0000	0.8519	1.0000	1	8
4	2 – 3	0.7647	1.0000	0.8283	1.0000	8	1
5	1 – 3	0.6941	1.0000	0.7803	1.0000	1	2
5	1 – 2	0.7024	1.0000	0.7932	1.0000	1	7
5	2 – 3	0.8286	1.0000	0.8819	1.0000	7	2

2 Supplementary Figures

Supplementary Figure 1. Stimulus selection process for the IMEI scale development.

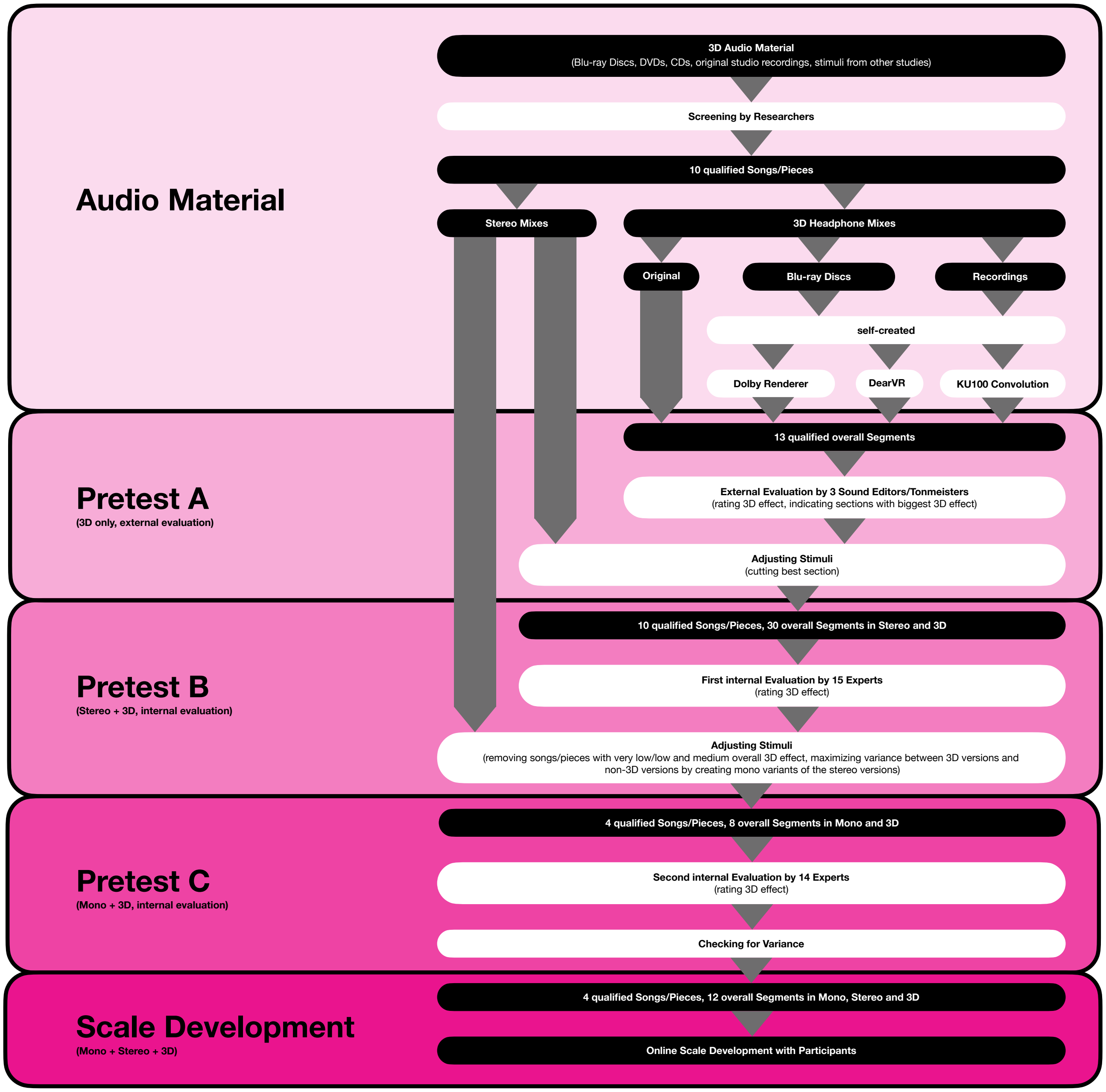

Supplementary Figure 2. Parallel analysis of the initial item set. Eigenvalues for the data and simulations are shown. The red line represents the Kaiser-Guttman criterion with an eigenvalue of 1.

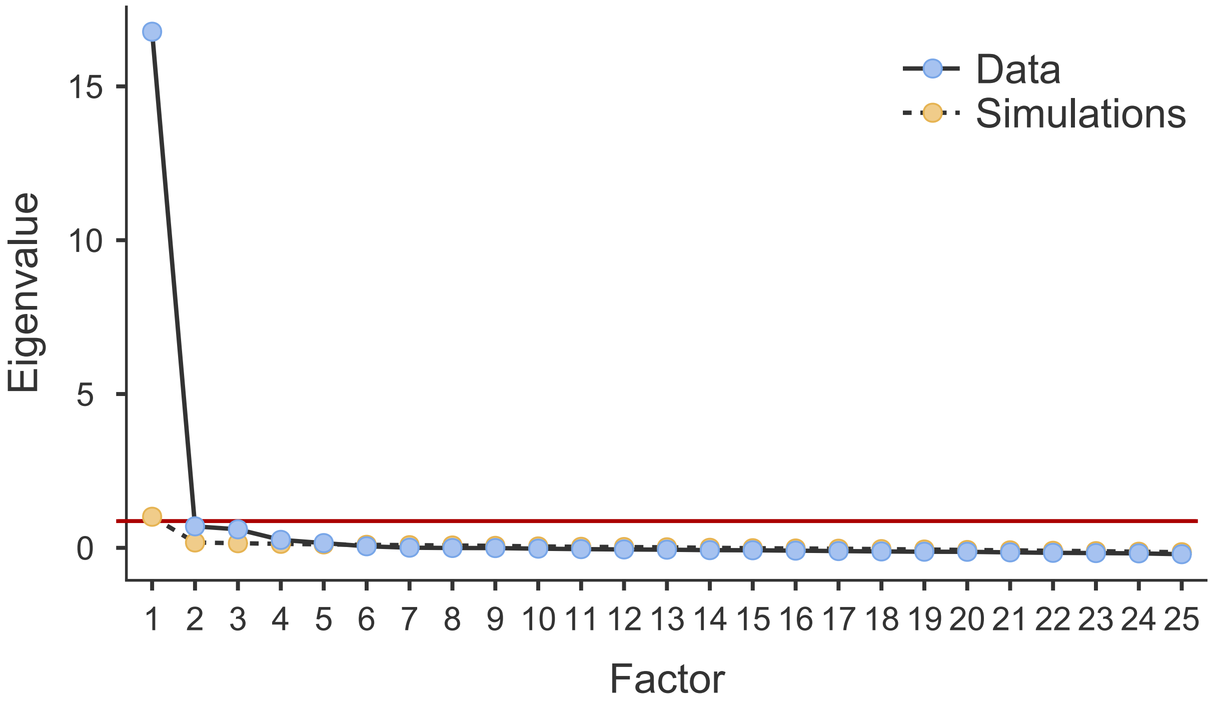

Supplementary Figure 3. Iterative procedure to identify the final item set for the Immersive Music Experience Inventory (IMEI). All statistics were provided by MFRM analysis.

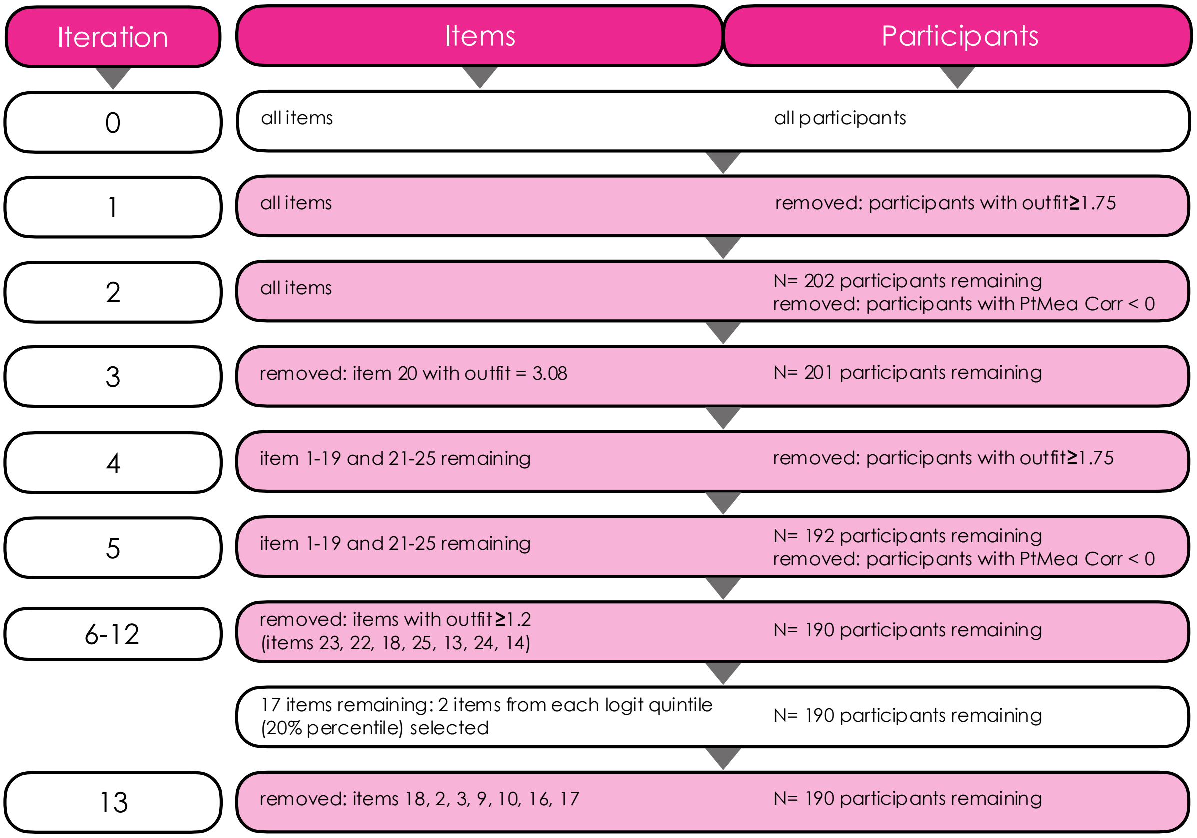

Supplementary Figure 4. Category probability curves of the MFRM analysis of the final item set. Curves show the model-based probability of observing a category of the response scale at each point on the latent variable *Immersion* relative to the item difficulty (output from Facets visualized with ggplot2 in R).

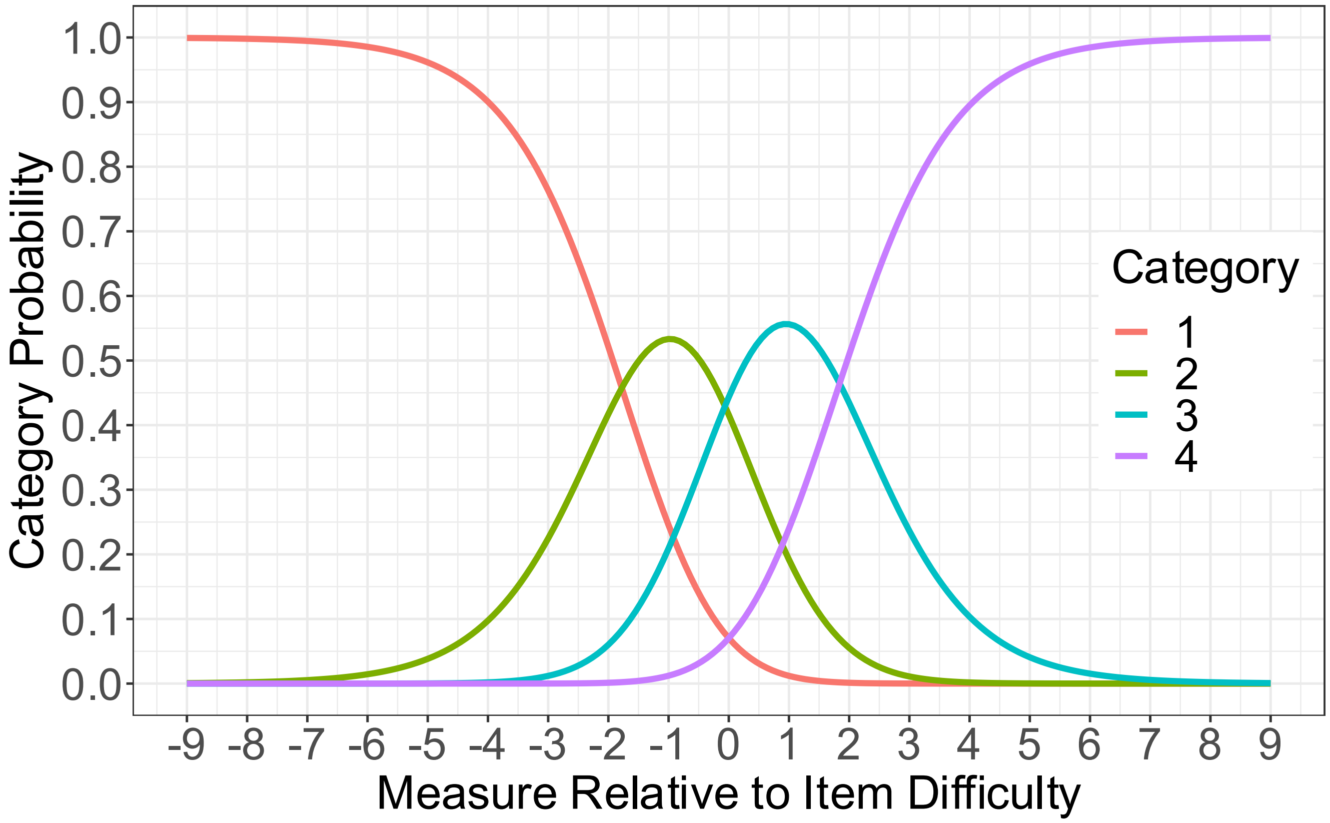

Supplementary Figure 5. Scatterplot for the mean IMEI score across all stimuli and the participant measure. $r(188) = .878$, 95% CI [.847, 1.0], $p < .001$ (one-tailed).

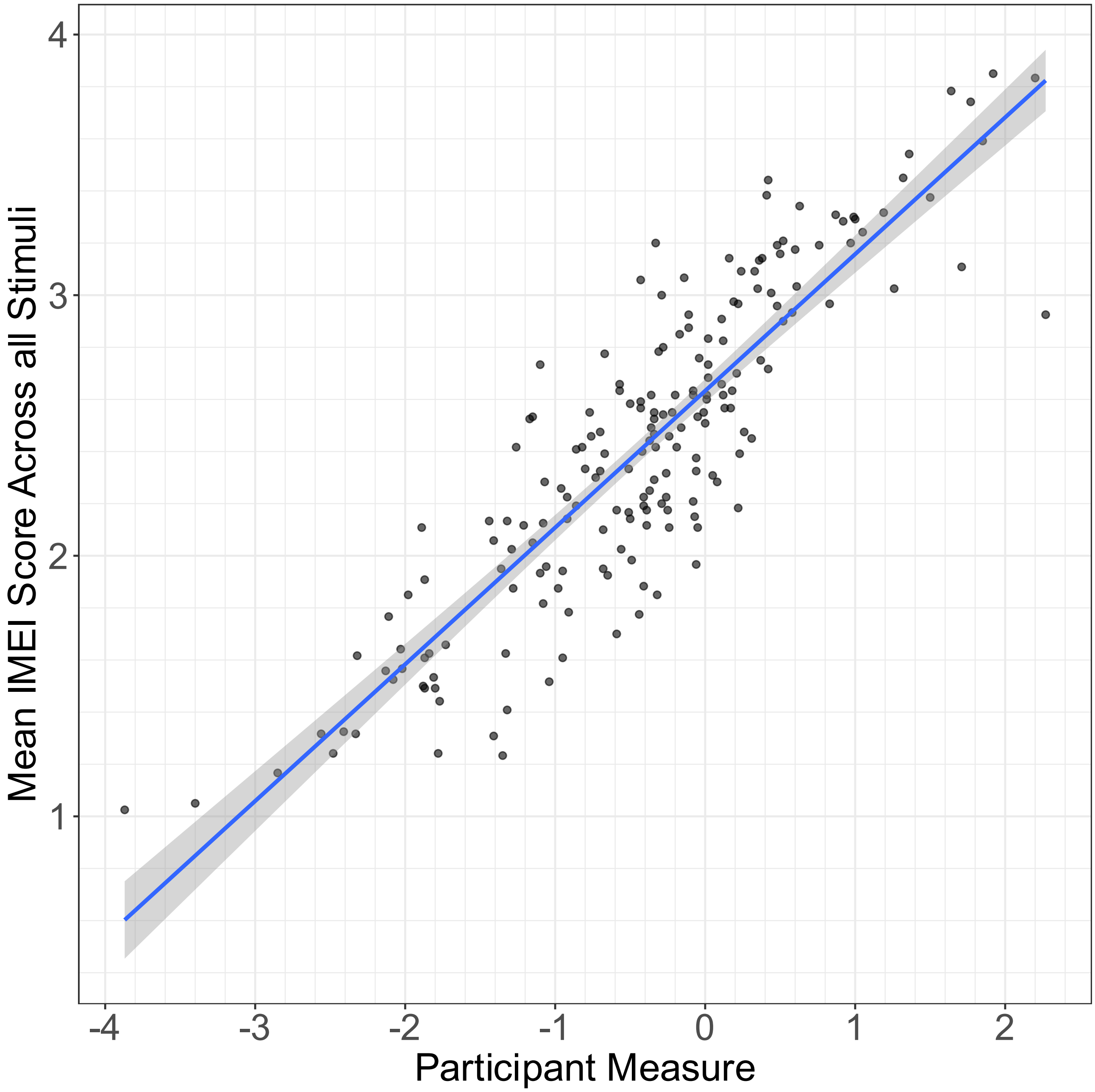

Supplementary Figure 6. Scatterplot for the IMEI score and the 3D impression. 3D impression was measured by item C2. $N = 2{,}280$ (190 participants × 12 stimuli) observations. Spearman's $\rho = .718$, $p < .001$ (one-tailed).

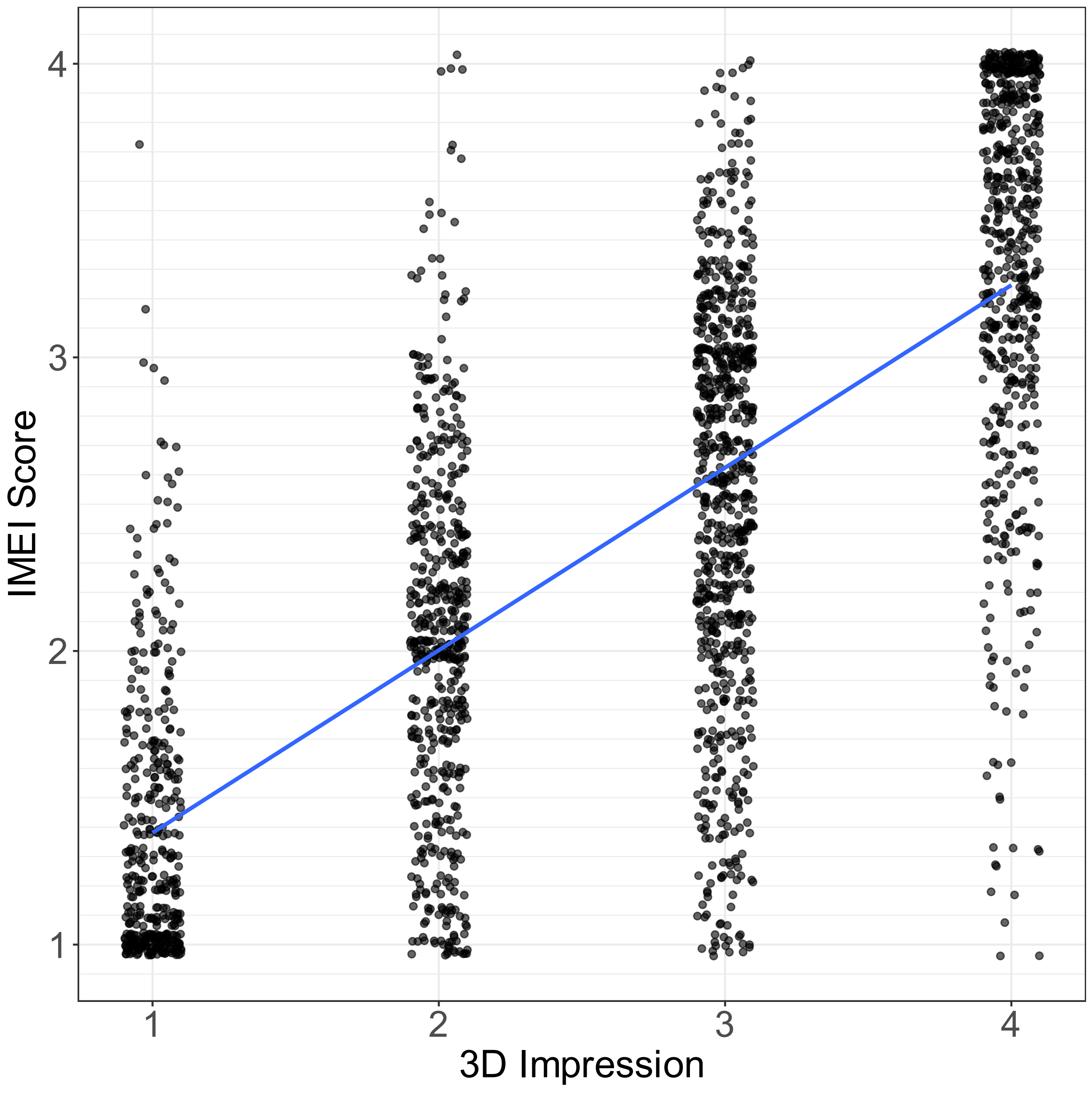

3 References

Georgiou, Y., & Kyza, E. A. (2017). The development and validation of the ARI questionnaire: An instrument for measuring immersion in location-based augmented reality settings. *International Journal of Human-Computer Studies*, *98*, 24–37. https://doi.org/10.1016/j.ijhcs.2016.09.014

Hartmann, T., Wirth, W., Schramm, H., Klimmt, C., Vorderer, P., Gysbers, A., Böcking, S., Ravaja, N., Laarni, J., Saari, T., Gouveia, F., & Maria Sacau, A. (2016). The spatial presence experience scale (SPES). *Journal of Media Psychology*, *28*(1), 1–15. https://doi.org/10.1027/1864-1105/a000137

Jennett, C., Cox, A. L., Cairns, P., Dhoparee, S., Epps, A., Tijs, T., & Walton, A. (2008). Measuring and defining the experience of immersion in games. *International Journal of Human-Computer Studies*, *66*(9), 641–661. https://doi.org/10.1016/j.ijhcs.2008.04.004

Lindau, A., Erbes, V., Lepa, S., Maempel, H.-J., Brinkman, F., & Weinzierl, S. (2014). A spatial audio quality inventory (SAQI). *Acta Acustica United with Acustica*, *100*(5), 984–994. https://doi.org/10.3813/AAA.918778

Reger, M. (1982). *Briefe an Fritz Stein* (S. Popp, Ed.). Carus.

Wagner, R. (2004). Das Bühnenfestspielhaus zu Bayreuth [The Bayreuth Festival Theatre]. In S. Friedrich (Ed.), *Digitale Bibliothek: Vol. 107. Richard Wagner: Werke, Schriften und Briefe. CD-ROM.* Directmedia Publishing. (Original work published 1873)

Witmer, B. G., Jerome, C. J., & Singer, M. J. (2005). The factor structure of the presence questionnaire. *Presence: Teleoperators and Virtual Environments*, *14*(3), 298–312. https://doi.org/10.1162/105474605323384654
